# Quasi-Static Deformations of Fiber-Reinforced Materials Based on Hyperelasticity

**DOI:** 10.3390/ma19101927

**Published:** 2026-05-08

**Authors:** Aleksander Franus, Stanisław Jemioło

**Affiliations:** Faculty of Civil Engineering, Warsaw University of Technology, Aleja Armii Ludowej 16, 00-637 Warsaw, Poland; stanislaw.jemiolo@pw.edu.pl

**Keywords:** fiber-reinforced materials, hyperelasticity, dissipative effects, quasi-static deformations

## Abstract

**Highlights:**

- Polyconvex-based formulation ensures existence of solutions in hyperelasticity.
- For moderately large deformations, the generalised Ciarlet model links parameters to the initial bulk modulus and Lamé’s constant.
- Scalar internal variable enables reversible description of damage-like processes.
- Transversely isotropic model with dissipative effects for fibre-reinforced solids.
- UMAT-based implementation supports finite element analysis in Abaqus.

**Abstract:**

This work addresses the quasi-static behavior of fiber-reinforced materials whose response is based on a hyperelastic formulation augmented by viscous and damage-like effects. A transversely isotropic constitutive model is developed within the framework of an internal scalar variable, enabling the reversible description of material damage while ensuring objectivity, thermodynamic admissibility and polyconvexity of the stored-energy function. The isotropic contribution is constructed from a generalized Ciarlet model, whereas the anisotropic part accounts for a family of elastic fibers embedded in a viscoelastic matrix, interpreted through a simple mixture theory. The resulting constitutive equations are implemented in Abaqus/Standard via a UMAT subroutine, and their rate form is derived consistently with the Zaremba–Jaumann objective stress rate. The performance of the model is examined by means of finite element simulations, including homogeneous tests in uniaxial strain and simple shear, relaxation and creep problems, and an inflation-like problem. The results demonstrate the capability of the model to capture strain-rate sensitivity, creep, stress relaxation and energy dissipation, as well as nonuniform deformation patterns, while highlighting its current limitation in representing permanent deformations.

## 1. Introduction

Materials exhibiting inelastic behavior are widely employed in engineering solutions within the field of civil engineering. Among the material types of interest are those sensitive to the rate of deformation and which, under standard service conditions, undergo finite elastic strains that exceed the range accurately captured by small-strain theory. These conditions apply, in particular, to elastomeric materials, utilized primarily as constituent components of bearings designed to attenuate vibrations. The work [1], for example, presents representative results of typical experimental tests performed on specimens of hard and soft elastomeric products. The loading path on the nominal stress–strain diagram does not coincide with the unloading path, indicating energy dissipation. Furthermore, loading–unloading cycles performed at different displacement-controlled deformation rates yield quantitatively distinct results. This material characteristic is referred to as viscosity [2], which is a property intrinsic to fluids but may equally pertain to solid bodies. An example of a structure exhibiting comparatively large deformations is a roof comprising a textile membrane. A structure of this type was analyzed in [3,4], employing fiber-reinforced hyperelastic material models, though without accounting for dissipative effects.

The rheological characterization of materials, that is, their sensitivity to the strain rate (viscosity) [5] and the Mullins effect [6], has been presented in various forms within the literature on the mechanics of solids undergoing large deformations [7,8]. The Mullins effect is typically represented by introducing a parameter that defines the maximum elastic energy attained during loading [6,9], or, alternatively, by a parameter that specifies the maximum deformation intensity [1,10]. This constitutes a particular case of a model formulated within the framework of internal variable theory [11,12], which provides a formal structure for incorporating inelastic effects in compliance with the second law of thermodynamics [13,14]. Another approach invokes the theory of materials with memory [15,16], in which approximations to the so-called response functional are introduced. One such approximation takes the integral form [17,18], leading to the class of quasi-linear viscoelastic (QLV) models [19,20]. Models of this type have also been developed for transversely isotropic and fiber-reinforced materials [21,22]. As conventional models describing the Mullins effect do not account for strain-rate sensitivity, they can be generalized, for example, within the QLV framework [23,24].

A dominant framework for fiber-reinforced hyperelasticity, particularly in the context of soft biological tissues and technical composites, is the Holzapfel–Gasser–Ogden (HGO) model [25], which represents the anisotropic contribution through pseudo-invariants associated with one or more fiber families. It should be noted, however, that the HGO model as implemented in standard finite element codes, including Abaqus/Standard [26], is formulated in terms of modified (deviatoric) invariants rather than the full invariants employed in the present work. This distinction has nontrivial consequences for volumetric–deviatoric coupling and for the behavior of nearly incompressible materials under finite strain. Extensions of the HGO framework to the finite-strain viscoelastic regime have been proposed by several authors [27]. The model developed herein may be viewed as a complementary formulation in which dissipative effects are carried entirely by the isotropic matrix and the full, unmodified invariants are preserved throughout.

Transversely isotropic QLV formulations have been developed in [21] or [23], among others. We note, however, that the formulation in [21] addresses rate-dependent viscoelasticity for transversely isotropic materials but does not incorporate damage or stress-softening effects of the Mullins type; it is, therefore, complementary to, rather than directly competing with, the present framework. The models [23,24] employ constitutive formulations—in terms of principal stretches and phenomenological softening functions, respectively—that differ structurally from the invariant-based, thermodynamically consistent internal-variable framework adopted here. A direct quantitative comparison between these approaches and the formulation presented here is meaningful only following careful parameter identification from the same experimental dataset. Such a comparison is, therefore, deferred to future work.

In the present work, attention is directed towards finite element implementation and solutions to selected boundary value problems employing the fiber-reinforced material model described in the authors’ previous works [28,29]. It is emphasized that the model satisfies the following requirements:

1. The requirement of objectivity associated with Galilean transformations and the given symmetry of the material in the initial configuration [30];
2. Thermodynamic admissibility through the restriction resulting from the Clausius–Duhem inequality [31];
3. Polyconvexity and growth conditions of the stored energy function [32].

The objectivity requirements in the context of constitutive relations pertain primarily to the function determining the stress tensor and the Helmholtz free energy function Ψ. Adopting the material description, the following conditions must hold:

(1) $$\begin{array}{cc} \Psi & =\hat{\Psi }\left(C,A\right),\\ T & =\bar{T}\left(X,t\right)=\hat{T}\left(C,A\right), \end{array}$$

where F is the deformation gradient tensor, $C=F^{T}F$ is the right Cauchy–Green tensor, A is a tensorial internal variable, and T is the second Piola–Kirchhoff stress tensor.

In addition to the objectivity requirement (Euler’s) related to motion and the observer of the current configuration, the objectivity requirement (Lagrange’s) of the material symmetry given in the initial configuration is also imposed. Therefore, the rotation tensor Q here refers to the rotation of the material coordinate system. We then say that the group G is a group of material symmetry if

(2) $$\Psi =\hat{\Psi }\left(C,A\right)=\hat{\Psi }\left(Q^{T}CQ,\;Q^{T}AQ\right),\;\forall Q\in G\subset SO\left(3\right).$$

When G=SO(3), the material is isotropic. Otherwise, we call the material anisotropic. We can describe the symmetry of the material through parametric tensors and, consequently, Ψ as a function of, e.g., anisotropic invariants of a symmetric second-order tensor [33,34]. If the parametric tensor is a symmetric second-order tensor $\bar{M}$ [35], then the symmetry group

(3) $$S_{o}=\left\{Q\in O\left(3\right)|\bar{M}=Q\bar{M}Q^{T}\right\}$$

defines an orthotropic material in the initial configuration of the body in its natural state [34,36].

In the present work, attention is restricted to the special case of a transversely isotropic material within the framework of hyperelasticity. The model of a transversely isotropic material contains a parametric tensor in the form M=m⊗m, where the unit vector m is consistent with the direction of the distinguished fibers of the material in the initial configuration [37,38]. Therefore, the stored energy function can be represented in the form of five invariants:

(4) $$\hat{W}\left(I_{i}\right)=\hat{W}\left(\mathrm{tr}C,\mathrm{tr}\mathrm{Cof}C,\det C,\mathrm{tr}\left(\mathrm{MC}\right),\mathrm{tr}\left(M\mathrm{Cof}C\right)\right).$$

From now on, we restrict ourselves to a purely mechanical theory, i.e., neglecting all thermal effects. Fields such as temperature and entropy are omitted in the balance equations. Nevertheless, such an approach is consistent with the second law of thermodynamics by introducing the so-called mechanical dissipation [30]. Therefore, the requirements resulting from the principles of thermodynamics are reduced to satisfying the Clausius–Duhem inequality [31] in the form

(5) $$D=\frac{1}{2}T\cdot \dot{C}-\Psi \geq 0.$$

Subsequently, determine the material time derivative of free energy with the introduction of one internal variable:

(6) $$\dot{\Psi }=\frac{\partial \hat{\Psi }}{\partial C}\cdot \dot{C}+\frac{\partial \hat{\Psi }}{\partial A}\cdot \dot{A}.$$

Substituting (6) into (5), we obtain

(7) $$D=\left(\frac{1}{2}T-\frac{\partial \hat{\Psi }}{\partial C}\right)\cdot \dot{C}-\frac{\partial \hat{\Psi }}{\partial A}\cdot \dot{A}\geq 0.$$

The inequality should hold for all admissible $\dot{C},\dot{\alpha }$. Therefore,

(8) $$T-2\frac{\partial \hat{\Psi }}{\partial C}{|}_{C=C^{T}}=0,\;D=-\frac{\partial \hat{\Psi }}{\partial A}\cdot \dot{A}\geq 0.$$

Upon substituting the tensorial internal variable with a scalar function *g*, the dissipation inequality reduces to

(9) $$\;D=-\frac{\partial \hat{\Psi }}{\partial g}\dot{g}\geq 0.$$

Apart from the requirements resulting from the second law of thermodynamics, objectivity, and material symmetry, additional requirements on the constitutive relations of hyperelasticity should be introduced so that a well-defined initial-boundary value problem of equations of motion has a solution [39]. Here, we primarily require the stored-energy function to be polyconvex and to satisfy appropriate growth conditions [32,40]. As the invariant $I_{4}=\mathrm{tr}\left(CM\right)$ is a convex function with respect to F∈Lin, while the invariant $I_{5}=\mathrm{tr}\left(M\mathrm{Cof}C\right)$ is a convex function with respect to CofF∈Lin, then the function takes the form

(10) $$\hat{W}\left(I_{i}\right)=W_{1}\left(I_{1}\right)+W_{2}\left(I_{2}\right)+W_{3}\left(I_{3}\right)+W_{4}\left(I_{4}\right)+W_{5}\left(I_{5}\right)$$

which is polyconvex if the functions $W_{1}\left(I_{1}\right)$ and $W_{4}\left(I_{4}\right)$ are convex functions with respect to the tensor F∈Lin, and the functions $W_{2}\left(I_{2}\right)$ and $W_{5}\left(I_{5}\right)$ are convex functions with respect to CofF∈Lin, while the function $W_{3}\left(I_{3}\right)$ is convex with respect to detF>0. It is known, however, that the condition is too strong for several reasons and should be replaced by a weaker condition than polyconvexity, namely, quasiconvexiy [41]. However, there is no known useful description of quasi-convexity under the local growth condition, i.e., W(F)→∞ as $\det F\to 0^{+}$.

This article is structured as follows: Section 2 develops the constitutive framework for fiber-reinforced hyperelastic materials with a scalar internal variable, including the material model and its quasi-static characterization. Section 3 describes the finite element implementation in Abaqus/Standard via a UMAT formulation and the associated rate-form constitutive equations. Section 4 presents numerical results for homogeneous tests and a nonuniform inflation-like problem, illustrating dissipation, rate effects, and instability-like responses. Section 5 summarizes the main conclusions and outlines directions for future work, particularly regarding permanent deformations and dynamic applications.

## 2. Fiber-Reinforced Material Models

### 2.1. General Framework

Fiber-reinforced material models are formulated by an additive decomposition of the Helmholtz energy [42,43] in the form

(11) $$\Psi =\left(1-\sum_{n=1}^{N}v_{n}\right)\Psi_{M}+\sum_{n=1}^{N}v_{n}\Psi_{Zn},\;N\leq 6,$$

The inelastic behavior is introduced through an internal variable represented by a scalar quantity, analogous to the constitutive relations of material models formulated within the framework of continuum damage mechanics (CDM) [44]. The principal distinction between standard CDM models and the model presented here lies in the ability to describe the reversibility of the material damage process [45]. We propose a Helmholtz free-energy function, $\Psi_{M}$, analogous to that adopted in [46], where wave-propagation problems were studied using the isotropic Murnaghan constitutive model. If we have

(12) $$\Psi_{M}=\hat{\Psi }\left(C,g\right)=\phi_{1}\left(g\right)\hat{W}\left(C\right)+\phi_{2}\left(g\right),\;C=\hat{C}\left(X,t\right),\;g=\hat{g}\left(C,t\right),$$

then, according to (9), we obtain

(13) $$D=\left(\frac{1}{2}T-\frac{\partial \hat{\Psi }}{\partial C}\right)\cdot \dot{C}+P\dot{g}\geq 0,$$

and

(14) $$T=2\phi_{1}\left(g\right)\frac{\partial \hat{W}\left(C\right)}{\partial C}{|}_{C=C^{T}},\;P=-\frac{\partial \hat{\Psi }\left(C,g\right)}{\partial g}=-\frac{\partial \phi_{1}\left(g\right)}{\partial g}\hat{W}\left(C\right)-\frac{\partial \phi_{2}\left(g\right)}{\partial g}.$$

We assume that the evolution equation of the variable *g* satisfies the inequality (13)

(15) $$\frac{\partial \phi_{1}\left(g\right)}{\partial g}\hat{W}\left(C\right)+\frac{\partial \phi_{2}\left(g\right)}{\partial g}+\alpha \dot{g}=0.$$

Indeed, if α>0 then

(16) $$D=P\dot{g}=\alpha {\dot{g}}^{2}\geq 0.$$

This clearly shows that both cases $\dot{g}>0$ and $\dot{g}<0$ can occur, which means that the scalar internal variable enables a reversible description of damage-like processes.

In the case of α=0 or α→+∞, the model does not predict dissipation, i.e., it describes a thermodynamically reversible process. From the thermodynamic requirements, it additionally follows that

(17) $$\left(\frac{\partial \phi_{1}\left(g\right)}{\partial g}\hat{W}\left(C\right)+\frac{\partial \phi_{2}\left(g\right)}{\partial g}\right){|}_{\dot{g}=0}=0.$$

We assume that $\phi_{1}>0$ and, furthermore, that the hyperelastic relation is recovered in the limit g=0. Therefore, $\phi_{1}\left(0\right)=1,\;\phi_{2}\left(0\right)=0$. Given the assumption of a natural state with zero dissipation as an initial condition t=0→C=I and g=0, (15) leads to

(18) $$\hat{W}\left(I\right)=0\Rightarrow \frac{\partial \phi_{2}\left(g\right)}{\partial g}{|}_{g=\hat{g}\left(I,0\right)=0}=0.$$

For this class of potentials, the structural requirements underlying the existence of weak solutions and physically realistic wave propagation may be stated concisely. The purely elastic contribution *W* is assumed to be polyconvex, and, hence, rank-one convex (strongly elliptic) in the domain of interest. The degradation function $\phi_{1}$ must be non-negative, convex, nonincreasing ($\phi_{1}^{\prime }\left(g\right)\leq 0$), and satisfy $\phi_{1}\left(0\right)=1$. The potential $\phi_{2}\left(g\right)$ is taken to be strictly convex, with $\phi_{2}\left(0\right)=\phi_{2}^{\prime }\left(0\right)=0$ and $\phi_{2}\left(g\right)\to \infty$ as *g* approaches the limit of the admissible interval from below. Under these conditions, the Helmholtz function remains polyconvex for each fixed *g* and convex in *g* for each fixed F, whilst coercivity is preserved by a barrier property of $\phi_{2}$. Moreover, as the instantaneous elastic stiffness scales with the positive factor $\phi_{1}$, the frozen-damage response inherits the rank-one convexity of *W*. Strong ellipticity is, therefore, retained at every admissible damage level. The rate-type evolution law for $\dot{g}$ supplies a viscous regularization that prevents the pathological localization frequently observed in rate-independent softening.

The function associated with the anisotropic part $\Psi_{Zn}$ of the energy is assumed to be in the form

(19) $$\Psi_{Zn}={\tilde{W}}_{Z}\left(I_{4n}\right),$$

where $I_{4n}=\mathrm{tr}\left(M_{n}C\right)$. As explained in the previous section of the article, the parametric tensors $M_{n}\equiv m_{n}⊗m_{n}$ are defined by the unit vectors $m_{n}$ associated with the distinguished directions of the fiber families. For n=1, function (11) leads to a simplified model of a transversely isotropic material [47,48,49].

The constitutive relations of the presented model classes can be interpreted as a composite material composed of a viscoelastic matrix and elastic fibers. It should be noted that in a similar way we could formulate equations describing a model with the interpretation of an elastic matrix and viscoelastic fibers.

### 2.2. Material Model

The function $W=\hat{W}\left(C\right)$ appearing in the previous equations pertains to hyperelasticity. Here, we adopt an isotropic function $W=\tilde{W}\left(I_{1},I_{2},J\right)$ in the form of a generalized Ciarlet model [50,51]:

(20) $$\hat{W}\left(C\right)=\tilde{W}\left(I_{1},I_{2},J\right)=a\left(I_{1}-3\right)+b\left(I_{2}-3\right)+c{\left(I_{1}-3\right)}^{2}+\beta \left(J\right)$$

where β is a convex function. From the assumption of a natural state, it follows that

(21) $$\frac{\partial \beta (J)}{\partial J}{|}_{J=1}=-2\left(a+2b\right).$$

In relation to the basic Ciarlet model, i.e., when c=0 [52], function (20) predicts a different qualitative response to a given deformation associated with shear. We assume the function β in the form

(22) $$\beta \left(J\right)=d\left(J^{2}-1\right)-2\left(a+2b+d\right)\ln J.$$

For parameter values a,b,d>0 and c≥0 the stored-energy function defines a polyconvex-based formulation that ensures the existence of solutions in hyperelasticity under the growth conditions.

If we consider moderately large deformations, the function (20) can be considered in a quadratic approximation with respect to the strain tensor E [52]. The model links parameters to the initial bulk modulus $K_{0}$ and Lamé’s constant $\lambda_{0}$ such that

(23) $$K_{0}=\frac{4}{3}\left(a+4b+6c+3d\right),\;\lambda_{0}=4\left(b+2c+d\right).$$

If we additionally assume that c=0 and

(24) $$a=\frac{1}{2}f\mu_{0},\;b=\frac{1}{2}\left(1-f\right)\mu_{0},\;f\in \left(0,1\right),$$

then we obtain

(25) $$\;d=\frac{1}{4}K_{0}-\frac{1}{3}\left(a+4b\right)>0.$$

We can also assume the parameter *c* in the form $c=h\mu_{0}>0$. Therefore, the model parameters can be represented by elastic constants. If we additionally assume that $K_{0}={\bar{k}}_{0}\mu_{0}$, then the stored energy function is scalable by $\mu_{0}$ and we can use a set of three dimensionless parameters $f,h,{\bar{k}}_{0}$, because

(26) $$\frac{a}{\mu_{0}}=\frac{1}{2}f,\;\frac{b}{\mu_{0}}=\frac{1}{2}\left(1-f\right),\;\frac{c}{\mu_{0}}=h,\;\frac{d}{\mu_{0}}=\frac{1}{12}\left(6f+3{\bar{k}}_{0}-24h-8\right).$$

The parameter values must satisfy the constraints

(27) $$f\in \left(0,1\right),\;{\bar{k}}_{0}>0,\;h\geq 0,\;6f+3{\bar{k}}_{0}-24h-8>0.$$

From the point of view of polyconvexity [35], we consider the function ${\tilde{W}}_{Z}$ as follows:

(28) $${\tilde{W}}_{Z}\left(I_{4}\right)=\frac{1}{4}E_{Z}{\left(I_{4}-1\right)}^{2},$$

where $E_{Z}>0$ can be interpreted as the initial Young’s modulus of the fiber family.

In view of the requirements established above, the functions $\phi_{1},\;\phi_{2}$ pertaining to the dissipative part of the material model may be taken [45] in the form

(29) $$\phi_{1}\left(g\right)=\left(1-g\right)\vartheta ,\;\phi_{2}\left(g\right)=-\frac{1}{2}\gamma \ln\left(1-g^{2}\right),\;\;g\in \left[0,1\right),$$

where the parameters γ,ϑ are positive scalars and γ has the dimension of energy per unit volume in the initial configuration. By analogy with the normalization employed for the Ciarlet model parameters, it proves expedient to introduce dimensionless parameters $\hat{\alpha }$ and $\hat{\gamma }$ for the moderately large deformation computations considered herein, defined through the relations $\alpha =\hat{\alpha }\mu_{0}\times \left[t\right]$ and $\gamma =\hat{\gamma }\mu_{0}$.

In the remainder of this work, the term “FRD model” shall denote the constitutive relation defined by the Helmholtz function (11) together with the fiber description (28). The dissipative part of the Helmholtz function within this model is understood as (12) in conjunction with the generalized Ciarlet model (20) and (29).

### 2.3. Quasi-Static Problem with Constant Deformation Rate

**Figure 1:**
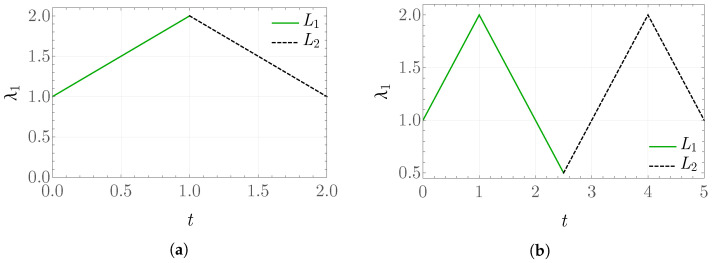
Prescribed stretch $\lambda_{1}$ as a function of time scale: (**a**) two intervals and (**b**) four internals.

To illustrate some fundamental characteristics of the model under consideration in the isotropic part, this subsection examines a quasi-static deformation problem with a constant deformation rate over time-scale intervals. The deformation type considered is triaxial compression/tension, such that $F\left(t\right)=\lambda_{1}\left(t\right)I$. In this case, the Cauchy stress tensor is also isotropic and is denoted as $\sigma \left(t\right)=\sigma_{11}\left(t\right)I$. The stretch ratio $\lambda_{1}$ is defined according to the plot shown in Figure 1.

The problem is solved assuming the parameter values $\mu_{0}=0.5\;\mathrm{MPa}$, f=0.35, h=0.1, and ${\bar{k}}_{0}=2$ or ${\bar{k}}_{0}=20$. The parameters pertaining to the dissipative part of the model are taken as $\hat{\alpha }=1$, $\hat{\gamma }=1$, ϑ=1. These values were selected to obtain less conventional solutions than those reported in the literature on damage mechanics [53,54]. As the evolution equation for the variable *g* is nonlinear, the solution is determined using the NDSolve procedure in the Wolfram Mathematica environment [55].

**Figure 2:**
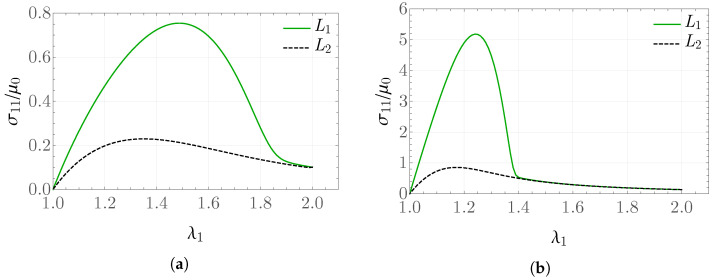
Scaled stress $\sigma_{11}/\mu_{0}$ for (**a**) ${\bar{k}}_{0}=2$ and (**b**) ${\bar{k}}_{0}=20$ in the problem with two intervals of constant deformation rate.

The plots in Figure 2 show the obtained values of the rescaled stress $\sigma_{11}/\mu_{0}=\hat{\sigma }\left(\lambda_{1}\right)$ for ${\bar{k}}_{0}=2$ and ${\bar{k}}_{0}=20$, respectively, in the case of a stretch defined on two intervals according to Figure 1a, that is, a single loading/unloading cycle. The pronounced difference in the results arises not only from the fact that, with increasing ${\bar{k}}_{0}$, a stiffer material response is modeled, but also because the larger values of elastic energy significantly affect the solution for the internal variable *g*.

**Figure 3:**
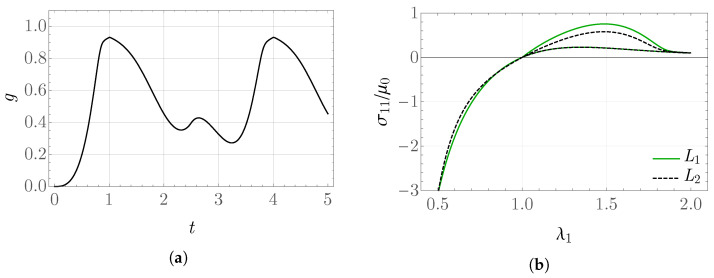
Internal variable (**a**) *g* and (**b**) $\sigma_{11}/\mu_{0}$ for ${\bar{k}}_{0}=2$ in the problem with piecewise constant rate.

**Figure 4:**
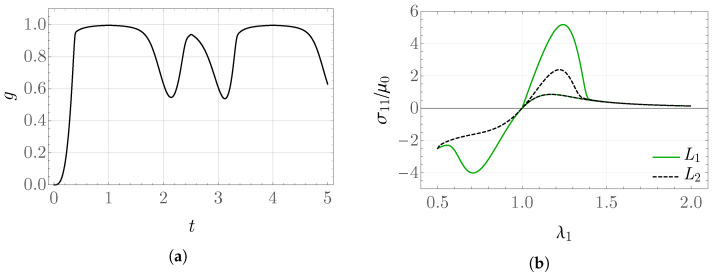
Internal variable (**a**) *g* and (**b**) $\sigma_{11}/\mu_{0}$ for ${\bar{k}}_{0}=20$ in the problem with piecewise constant rate.

The obtained values of the internal variable *g* and the rescaled stress $\sigma_{11}/\mu_{0}$ as functions of $\lambda_{1}$ for the prescribed stretch shown in Figure 1b are presented in Figure 3 and Figure 4. From the form of the evolution equation for the variable *g*, it is known that the solution depends directly on the value of the stored-energy function, which can be observed in the behavior of the plot of *g*. For ${\bar{k}}_{0}=20$, the values of elastic energy are significantly larger than for ${\bar{k}}_{0}=2$, which results in a much greater reduction in the obtained stress between the values in the time intervals $L_{1}$ and $L_{2}$. This may be interpreted as a more pronounced damage of the modeled material.

## 3. Material Model Implementation

### 3.1. Abaqus/Standard User Subroutines

Abaqus provides multiple means of implementing constitutive relations for user-defined material models [56]. The primary tools are the so-called user subroutines, written in Fortran or C/C++, which interact with the core Abaqus module through well-defined interfaces. These subroutines may either modify standard procedures or introduce, among other things, custom material models. From the standpoint of hyperelasticity, the subroutines UHYPER and UANISOHYPER_INV are of particular relevance, as they enable the definition of constitutive relations for isotropic and anisotropic materials by specifying the stored energy density function together with its derivatives with respect to the appropriate deformation invariants [57]. On the basis of these quantities, the program determines the components of the Cauchy stress tensor σ and the components of the tangent stiffness tensor $C_{B}$, accounting for the objectivity requirement.

(30) $$\dot{\tau }=J\left(W\sigma +\sigma W^{T}+C_{B}\cdot D\right).$$

In Abaqus/Standard, the Zaremba–Jaumann rate is employed [26]. For the Kirchhoff stress tensor, one has

(31) $$\dot{\tau }=\;\overset{\circ }{\tau }+W\tau -\tau W=\;\overset{\circ }{\tau }+L\tau +\tau L^{T}-\left(D\tau +\tau D\right).$$

For constitutive relations incorporating dissipative effects, the UMAT subroutine interface is employed. It requires the definition of the components of the Cauchy stress tensor and the components of the tangent stiffness tensor ∂Δσ/∂Δϵ arising from the constitutive relation. If the tangent stiffness tensor is understood as $C_{B}$ in (30), the implementation is consistent with the hyperelastic equations, and such an approach is referred to as the total formulation [26]. In this case, finite elements with a mixed formulation may be employed, though the definition of appropriate third-order derivatives is required in a manner analogous to the UHYPER procedure.

### 3.2. Rate Form of Constitutive Equations

The incremental form of the constitutive relation of FRD model, incorporating the Zaremba–Jaumann objective rate, may be expressed as

(32) $$\overset{\circ }{\tau }\;=C\cdot D=\phi_{1}\left(g\right){\overset{\circ }{\tau }}_{M}+{\dot{\phi }}_{1}\left(g\right)\tau_{M}+{\overset{\circ }{\tau }}_{Z},$$

where

(33) $${\overset{\circ }{\tau }}_{M}=\left(1-\sum_{n=1}^{N}v_{n}\right){\bar{C}}_{M}\cdot D,\;{\overset{\circ }{\tau }}_{Z}=\sum_{n=1}^{N}v_{n}C_{Z_{n}}\cdot D,\;N\leq 6.$$

The tangent stiffness tensors ${\bar{C}}_{M},\;C_{Zn}$, associated with the generalized Ciarlet model and the polynomial of the invariant $I_{4n}$, were determined, for example, in [43]. Consequently, the evaluation of tensor C in (32) additionally requires the determination of the expression ${\dot{\phi }}_{1}\left(g\right)\tau_{M}$. From the adopted form of the evolution equation for the internal variable, it follows directly that $\dot{g}$ may be treated as a function of the variable $W_{M}$. It, therefore, follows that

(34) $$\begin{array}{cc} \; & {\dot{\phi }}_{1}\left(g\right)\tau_{M}=\frac{\partial \phi_{1}}{\partial g}\frac{\partial g}{\partial W_{M}}{\dot{W}}_{M}\tau_{M}=\left[g_{W}\phi_{1}^{\prime }\left(g\right)\tau_{M}⊗\tau_{M}\right]\cdot D,\\ \; & g_{W}\equiv \frac{\partial g}{\partial W_{M}},\;\phi_{1}^{\prime }\left(g\right)\equiv \frac{\partial \phi_{1}\left(g\right)}{\partial g}, \end{array}$$

together with

(35) $$\phi_{1}^{\prime }\left(g\right)+\alpha {\dot{g}}_{W}=0,\;W_{M}=\left(1-v\right)W_{0},\;v=\sum_{n=1}^{N}v_{n}.$$

The implementation of Equation (35) is carried out by applying the forward Euler method over integration steps $s_{n+1}=s_{n}+\Delta s$, employing

(36) $$g_{W,k+1}=g_{W,k}-\frac{\Delta s}{\alpha }\phi_{1,k}^{\prime }.$$

If $\phi_{1}\left(g\right)=1-g$, the derivative $\phi_{1}^{\prime }$ is constant, namely, $\phi_{1}^{\prime }\left(g\right)=-1$. In this case, a straightforward relation is obtained

(37) $$g_{W,k+1}=g_{W,k}+\frac{\Delta s}{\alpha }.$$

The complete constitutive model, including the dissipative contribution associated with the scalar internal variable, is implemented in Abaqus/Standard via a UMAT subroutine and used in the finite element study presented in the following section.

## 4. Results

### 4.1. Numerical Validation

#### 4.1.1. Hyperelasticity

The correctness of the implementation of the constitutive relation of FRD model via the UMAT subroutine was initially verified through uniaxial strain and simple shear tests on a single C3D8R finite element, without accounting for inelastic effects. All boundary conditions were specified as displacement-controlled. The parameters of the hyperelastic model were adopted as $\mu_{0}=0.5\;\mathrm{MPa},\;k_{0}=2\mu_{0},\;f=0.7,\;h=0.1,v=0.05,E_{z}/\mu_{0}=10$.

**Figure 5:**
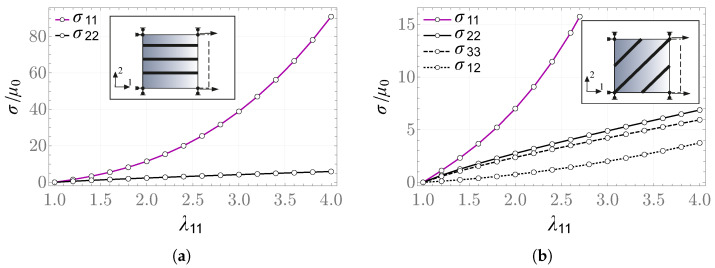
Uniaxial strain problem: (**a**) fiber direction $m=b_{1}$, (**b**) fiber direction $m=1/\sqrt{2}\left(b_{1}+b_{2}\right)$.

**Figure 6:**
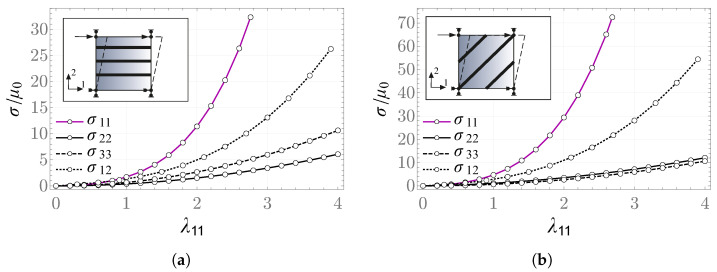
Simple shear problem: (**a**) fiber direction $m=b_{1}$, (**b**) fiber direction $m=1/\sqrt{2}\left(b_{1}+b_{2}\right)$.

The computed stress values are in agreement with those obtained from the analytical expressions, within the limits of the prescribed numerical accuracy. Figure 5 and Figure 6 present plots of the rescaled components of the Cauchy stress tensor.

#### 4.1.2. Fiber-Reinforced Model with Dissipative Effects

The next step in the verification of the implementation of FRD model is the inclusion of viscous effects. To this end, relaxation and creep tests were carried out, adopting in the former the parameter values $\hat{\alpha }=1,\;\hat{\gamma }=1$, and in the latter $\hat{\alpha }=0.5,\;\hat{\gamma }=0.2$.

The relaxation problem concerns a uniaxial strain state with displacement boundary conditions. The deformation gradient tensor is taken in the form $F=2\;b_{1}⊗b_{1}\;H\left(t\right)$, and the fiber direction as $m=\frac{1}{\sqrt{2}}(b_{1}+b_{2})$. In the creep problem, a plane stress is considered, i.e., the stress state is defined by the tensor $\sigma =\sigma_{11}\;b_{1}⊗b_{1}$, and the deformation state by $C={\left(\lambda_{11}\right)}^{2}b_{1}⊗b_{1}+{\left(\lambda_{22}\right)}^{2}\left(b_{2}⊗b_{2}+b_{3}⊗b_{3}\right)$. It is assumed that the “11” stress component is controlled by prescribing the traction vector on the undeformed surface of the finite element, $t_{s}=2\mu_{0}H\left(t\right)b_{1}$.

**Figure 7:**
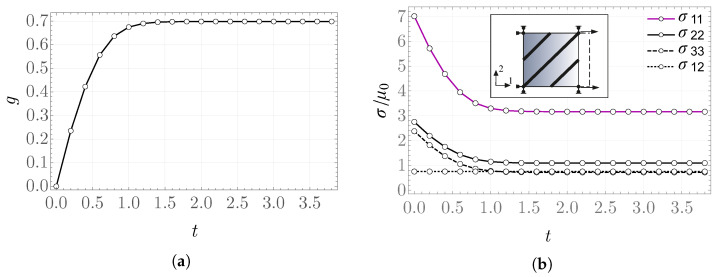
(**a**) Internal variable and (**b**) nonzero components of the Cauchy stress tensor as functions of time scale in the relaxation problem—FRD model.

Figure 7 presents plots of the internal variable and the rescaled, nonzero components of the Cauchy stress tensor in the relaxation problem. It is worth noting that the value of the $\sigma_{12}$ component does not change over the considered time scale, as the constitutive relation in the anisotropic part does not provide for relaxation.

**Figure 8:**
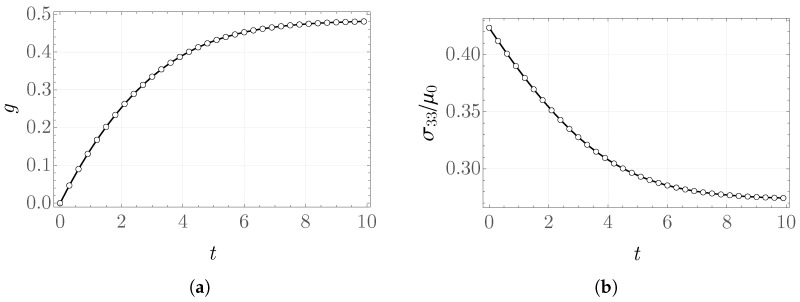
(**a**) Internal variable and (**b**) scaled stress $\sigma_{33}/\mu_{0}$ as a function of time scale in the creep problem—FRD model.

**Figure 9:**
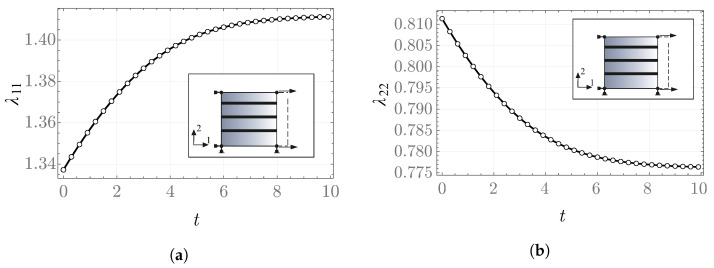
Stretches (**a**) $\lambda_{11}$, (**b**) $\lambda_{22}$ as functions of time in the creep problem—FRD model.

In the creep problem, the fiber direction is taken to be aligned with the vector $m=b_{1}$. The resulting values of the internal variable and the stretches as functions of time are shown in Figure 8 and Figure 9. The results obtained with Abaqus are consistent with those computed by means of the NDSolve procedure in Mathematica.

### 4.2. Nonuniform Deformations

**Figure 10:**
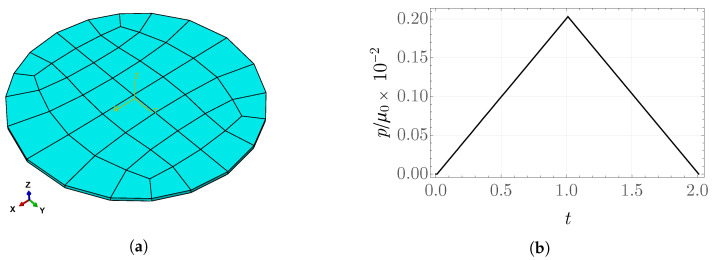
(**a**) Finite element mesh; (**b**) rescaled pressure $p/\mu_{0}$ in the inflation problem.

An inflation-like problem is considered for a geometry in the form of a circle with radius *R* and thickness R/20, subject to the boundary conditions $u_{2(y)}=0,\;u_{3(z)}=0$. The analysis is carried out for the FRD model with the parameter values $\hat{\alpha }=0.2,\;\hat{\gamma }=0.01,$ $\mu_{0}=0.5\;\mathrm{MPa},\;{\bar{k}}_{0}=2,\;f=0.7,\;h=0.1,\;v=0.05,\;E_{z}/\mu_{0}=10$. The fiber family direction is assumed to be aligned with the *x*-direction. As the implemented UMAT procedure is dedicated to a three-dimensional stress state, a rectangular, eight-node, reduced-integration brick element of type C3D8R is employed. This element type is not associated with a shell-theory formulation [58]. The loading and unloading are applied by prescribing a pressure on the upper surface of the circle, as illustrated in Figure 10. The discretized domain consists of 44 finite elements.

**Figure 11:**
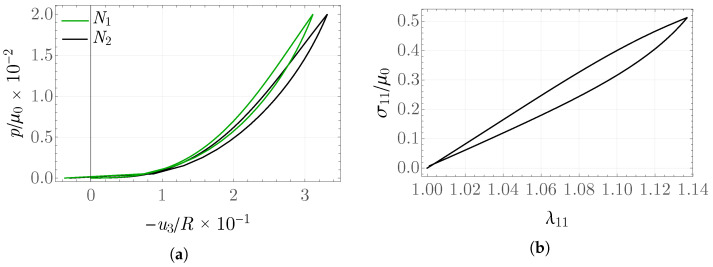
(**a**) Normalized pressure $p/\mu_{0}$ as a function of midpoint displacement; (**b**) rescaled stresses $\sigma_{11}/\mu_{0}$.

**Figure 12:**
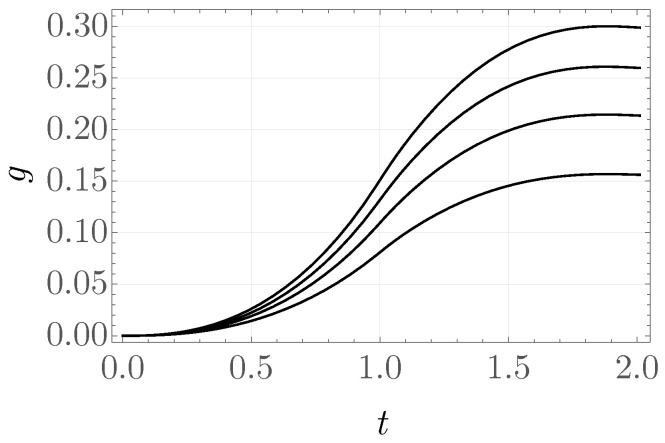
Internal variable as a function of time scale, evaluated in selected finite elements.

Figure 11 shows plots of the normalized pressure as a function of the displacement of the region midpoint. It is worth noting that, in the final unloading phase, a “jump” in the displacement value occurs to positive values, where the next cycle of loading is also presented. It may be interpreted as a form of instability; see [59]. The rescaled stress values $\sigma_{11}/\mu_{0}$ as a function of stretch in the loading–unloading process are presented in Figure 11. These values were extracted from one of the central elements and clearly indicate energy dissipation. The internal variable, evaluated for several selected elements, attains maximum values between 0.15 and 0.30; see Figure 12.

## 5. Conclusions

A proposed generalization of the constitutive relations of hyperelastic models, incorporating fiber reinforcement and viscous effects, involves the introduction of an internal variable in the form of a scalar quantity, analogous to the constitutive relations of material models formulated within the framework of continuum damage mechanics (CDM). These models belong to the class of fiber-reinforced material models [38], formulated additionally within the framework of a simple mixture theory [42]. The form of the isotropic component of the Helmholtz free-energy function was adopted from [45] and subsequently extended to encompass the generalized Ciarlet model and fiber reinforcement, yielding a transversely isotropic material model incorporating dissipative effects [28]. The principal distinction between CDM models and the class of models presented in this work lies in the ability to describe the reversibility of the material damage process. As demonstrated in the boundary-value problems discussed herein, besides modeling the phenomenon of material damage, the constitutive relations within this class also capture strain-rate sensitivity as well as creep and stress-relaxation phenomena. The principal limitation of the models presented lies in their inability to capture permanent deformations. This aspect, together with the investigation of dynamic effects in initial-boundary value problems based on the proposed material model, will be addressed in future work.

## Data Availability

The original contributions presented in this study are included in the article. Further inquiries can be directed to the corresponding author.
